# Case Report: Direct Visualization of the Nervus Intermedius During the Microvascular Decompression Procedure: Should We Take It Seriously?

**DOI:** 10.3389/fneur.2021.666427

**Published:** 2021-06-09

**Authors:** Rui-zhe Zheng, Chang-yi Zhao, Zhi-jie Zhao, Xin-yuan Li

**Affiliations:** Department of Neurosurgery, Tongren Hospital, Shanghai Jiao Tong University School of Medicine, Shanghai, China

**Keywords:** nervus intermedius neuralgia, microsurgical decompression, treatment, surgical outcome, facial paralysis

## Abstract

**Background:** Nervus intermedius neuralgia (NIN), known as geniculate neuralgia (GN), is an uncommon cranial nerve disease caused by an offending vessel compressing the nervus intermedius (NI). Microvascular decompression (MVD) has now become a valued treatment approach for NIN because it can resolve neurovascular conflict (NVC) at the root entry zone of the NI. In the era of continuously optimizing and improving the surgical technique of MVD, further minimization of all possible postoperative complications is not only welcome but also necessary.

**Objective:** The aim of this work is to assess the postoperative outcome of direct visualization of the NI during the MVD procedure.

**Methods:** This study retrospectively reviewed the clinical records of a group of seven consecutive patients with NIN who underwent MVD in the period of 2013–2020 in our clinic and 16 studies reported NIN patients who underwent MVD in the period of 2007–2020.

**Results:** In total, 91.3% of all patients experienced immediate and complete relief of cranial neuralgia after MVD. Six of 23 patients have experienced direct visualization of the NI intraoperatively, and 66.7% of those patients had complications such as facial paralysis, dysacousia, or a combination of these conditions postoperatively. Slight surgical approach-related complications such as complaints associated with excessive drainage of cerebrospinal fluid (CSF), nausea and vertigo, and delayed wound union were observed in 80% of the remaining 15 patients, and these symptoms are totally relieved in the telephone and outpatient follow-up after 6 months.

**Conclusion:** Our case series shows that MVD produced immediate pain relief in the majority of NIN patients. MVD carries surgical risk, especially in patients who experience direct visualization of the NI after mechanical stretch and blunt dissection in surgical procedures. Attempts to avoid mechanical stretch and blunt dissection of the compressed nerve were important for intraoperative neuroprotection, especially facial nerve protection

## Highlights

- Dissection to direct visualization of the NI caused postoperative facial paralysis after MVD.
- There was no need to directly dissect out the NI while all obvious compressing vessels were separated from the VII/VIII cranial nerve complex.
- Attempts to avoid mechanical stretch and blunt dissection of the compressed nerve were important for intraoperative neuroprotection, especially facial nerve protection.

## Introduction

The nervus intermedius (NI) was first identified by Eustachius in 1563 and was clearly described by Wrisberg in 1777 (1). This nerve contains three types of nerve fibers: (1) gustatory sensory fibers, which originate from the receptors of the anterior two-thirds of the tongue and project to the superior pole of the solitary nucleus in the medulla; (2) cutaneous sensory fibers, which originate from the external auditory canal (EAC) and project to the dorsal side of the spinal trigeminal tract; and (3) preganglionic parasympathetic fibers, which originate from the superior salivary nucleus and project to the pterygopalatine and submandibular ganglions, supplying lacrimal, sublingual, submandibular, and accessory salivary glands and glands of the nose and palate, respectively (2–5). To our knowledge, cutaneous sensory fibers play an important role in primary otalgia (6).

Nervus intermedius neuralgia (NIN), known as geniculate neuralgia (GN) and characterized by neuralgic pain deep inside the ear, is a rare and idiopathic condition of otalgia. Three of the following four characteristics form a clinical diagnosis of NIN: (1) paroxysm by stimulation of the posterior wall of the EAC or periauricular region, (2) symptoms lasting seconds to minutes for each attack, (3) pain with severe intensity, and (4) pain that is stabbing or fulgurant in nature (7). To date, no consensus has been reached on the precise pathophysiology of such disease [entities such as viral infection (herpes) and Ramsay Hunt syndrome] (8–10). However, the most widely accepted opinion is that NIN is caused by vascular compression of the NI in the entry zone of the brainstem, which is a mechanism similar to that of other cranial nerve neuralgias (2). At this point, microvascular decompression (MVD) seems to be a beneficial surgical choice for the management of NIN.

Previously, it had long been thought that the NI is entirely related to VII. However, Rhoton et al. found that there was independence between the NI and VII, and the average region of adherence with VII was only 8 mm (4). Such authoritative findings have been taken as the anatomical basis for the subsequent surgical procedure. Some surgeons have tried various strategies to treat NIN, such as the previously proposed NI transection and the recently advocated MVD (11, 12). Despite this fact, there are limited guidelines on different surgical approaches in specific scenarios, and this motivates our interest in sharing our experience with treating NIN by MVD. In this retrospective study, we detail the patient characteristics, the preoperative imaging examination, and especially the necessary surgical skills.

## Methods

### Study Design

We performed a retrospective analysis of patients with NIN who underwent MVD in the period of 2013–2020 in our hospital. All patients with NIN were diagnosed, treated, and followed-up in our department. The criteria included NIN patients who (1) met the *International Classification of Headache Disorders, Third Edition* (ICHD-3), diagnostic criteria (13); (2) had no congenital cranial nerve diseases, primary adult (>18 years) onset NIN and secondary symptoms such as complications of Herpes zoster or intracranial space-occupying lesion; (3) had no previous surgical history of craniocerebral disease; (4) had complete case information during hospitalization; and (5) provided routine informed consent and consent for data presentation.

Diagnosis and treatment were performed by a professional cranial nerve treatment team that included at least one neurosurgery specialist with a senior professional title. In order to correctly diagnose NIN, we use a specific and elaborate diagnostic work-up. At first, primary ear disease and referred otalgia were excluded by otoscopy and laryngopharyngoscopy. Secondly, magnetic resonance imaging (MRI) of the head region was performed to exclude alternative pathologies and thus to rule out the other causes of secondary otalgia. Then, a 3.0-Tesla magnetic resonance tomographic angiography (MRTA) examination was used to identify NVCs involving the VII/VIII cranial nerve complex. If conservative pain management did not lead to adequate pain reduction, patients were eligible for MVD. In our hospital, a threshold to opt for MVD surgery was patients either did not have sufficient relief from conventional therapies, suffered side effects of the medication (e.g., carbamazepine and gabapentin), or obvious deformity of the VII/VIII complex resulting from severe compression of the small vessels in the MRTA imaging. Finally, all patients identified with NIN underwent MVD. Postoperatively, patients were followed-up and treated until they reached the discharge standards, and then, they were followed up for 6 months.

This study was approved by the ethics committee of our institution. Written informed consent was obtained from the individual(s) for the publication of any potentially identifiable images or data included in this article.

### Surgical Procedure

A routine MVD via a retrosigmoid approach was performed to treat our patients (11). Sufficient cerebrospinal fluid (CSF) was released to minimize cerebellar traction and obtain enough operating space. Then, the pontomedullary sulcus was visualized, and the relationship between the vessel and cranial nerve was studied after the arachnoid membrane around the nerves was opened. All cranial nerves that proved to be compressed before the operation were carefully identified and explored as needed (the order of microscopic view transfer was first to the posterior cranial nerves, then to the facial–acoustic nerve complex, and finally to the trigeminal nerve). It is noteworthy that sharp or blunt dissection until direct visualization of the NI was performed and mainly depended on whether there was a clear gap or obvious adhesion between the facial and auditory nerves. Repeated separation was able to be avoided, while all the adhesive arachnoids surrounding the VII/VIII nerve complex were sharply opened. After the offending vessel was moved away from the compressed nerve, a small piece of Teflon sponge was placed in the narrow gap. Meanwhile, we transferred a permanent sling suspension to the petrous dura in a caudo-antero-lateral direction to maintain full decompression. Finally, the dura mater was sutured in a watertight pattern, and the surgical wound was routinely closed.

### Clinical Data and Follow-Up

Patient baseline characteristics were collected from the medical records in our hospital, and patients were classified based on potential underlying cranial neuralgia and the surgeon's operative note (e.g., NIN: nervus intermedius neuralgia/TN: trigeminal neuralgia/GPN: glossopharyngeal neuralgia/HFS: hemifacial spasm). Preoperative information included age at presentation and treatment, duration of symptoms, relevant history, and the nature and range of symptoms. The other pathologies were ruled out by a board of certified radiologists and neurologists, and vascular compression involving the VII/VIII cranial nerve complex was presented in MRTA. Operative findings were analyzed based on detailed operative records. Postoperative follow-up was performed until the discharge criteria were reached, and we conducted telephone and outpatient follow-up for 6 months after discharge. Excellent outcome was defined as total relief of NIN symptoms, no significant complications at discharge, and without permanent complications.

### Literature Review

Systematic review of the previous literature was carried out by searching PubMed, MEDLINE, Embase, and Google Scholar databases since 2007 (there were no surgical recipients of MVD for NIN prior to 2007 (2)), using MeSH/keywords “geniculate neuralgia” or “nervus intermedius neuralgia” together with “microvascular decompression.” Non-English language publications and articles not recording detailed surgical information (e.g., reviews, meta-analyses, or perspectives) were excluded. Included literatures were reviewed in detail to confirm the diagnosis of NIN or GN, to capture data points including total case size; presenting symptoms; other cranial neuralgias; surgical information; operative approach; and, especially, intraoperative findings, temporary and follow-up outcomes, and total follow-up duration.

## Results

### Preoperative Clinical Data

This retrospective study group finally consisted of seven patients (three males, four females; age range, 22–73 years) from our tertiary medical center in the period of 2013–2020. In all patients, the typical symptoms of pain predominantly located in the deep side of the EAC, paroxysmal attacks precipitated by stimulation and lasting seconds to minutes, and pain intensity that varied greatly met the diagnostic criteria of NIN. Before presentation, those patients had been medically treated with carbamazepine, ibuprofen, gabapentin, or a different combination of these treatments.

**Figure 1:**
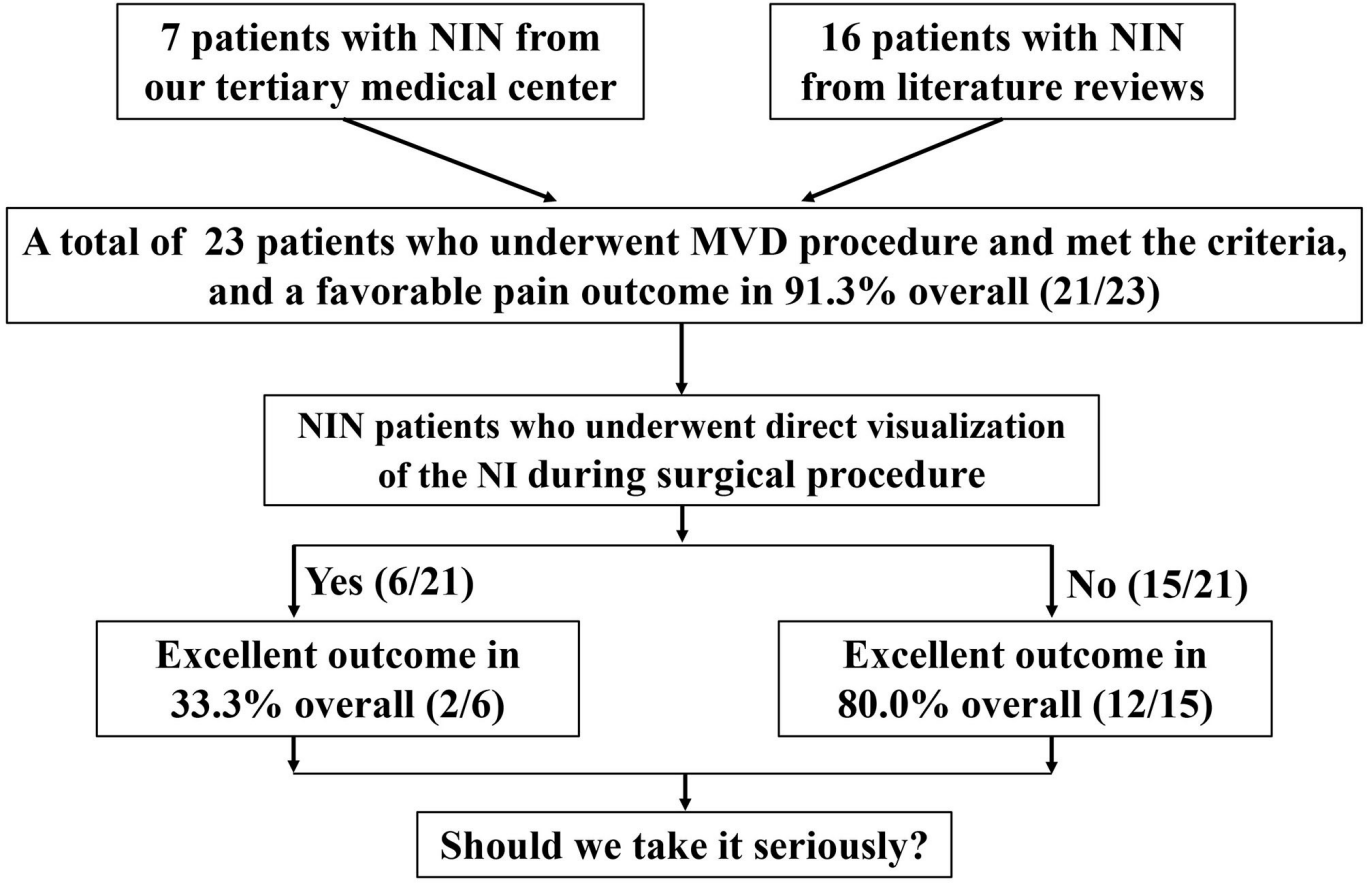
A flowchart of the recruitment and clinical trajectories for 23 patients who underwent MVD.

In addition, there was a total of 16 patients (3 males and 13 females; age range, 17–76 years) from the identified 9 candidate literatures in the period 2007–2020. Similarly, they meet the diagnostic criteria of NIN, and those patients have experienced different pharmacological treatments (e.g., carbamazepine, amitriptyline, pregabalin, gabapentin, ibuprofen, anesthetic blocks, or a different combination of these treatments). All the preoperative baseline characteristics are displayed separately in Table 1. A flowchart of the recruitment and patients' clinical trajectories is diagrammed in Figure 1.

**Table 1:** Patients' baseline characteristics.

**Patient**	**Sex**	**Onset**	**Surgery**	**Previous management**	**Symptoms and region**	**History**
		**Years**				
**Our cases**						
Case 1	F	20	22	Ibuprofen and carbamazepine	Left EAC and radiating to the parietal region	None
Case 2	F	33	37	Carbam azepine	Deep inside the right EAC	None
Case 3	M	49	50	Ibuprofen	Deep side of the left EAC and radiating to the left maxillofacial region	Left TN and GPN
Case 4	F	71	73	Carbamazepine	Deep inside the left EAC radiating to the posterior alveolar region	Left TN
Case 5	M	41	46	Brief history of ibuprofen	Deep inside the left EAC and auricle	Middle ear infection in childhood
Case 6	M	40	51	Carbamazepine	Deep inside the left EAC	Right HFS
Case 7	F	60	66	Carbamazepine and gabapentin	Deep inside the right EAC and radiating to the perimastoid region	Maxillary sinusitis
**Cases reported in the literatures**						
Onoda et al. (8)	M	48	68	Carbamazepine	Left EAC and radiating to the parietal region	Left tinnitus and HFS
Inoue et al. (14)	F	26	36	Carbamazepine	Deep inside the left EAC	None
Goulin Lippi Fernandes et al. (2) 8 cases, 2007–2015	F	32	35	Carbamazepine and amitriptyline	Deep inside the EAC and radiating to ipsilateral face	Maxillary sinusitis
	F	17	18	Amitriptyline	Bilateral pain deep inside of the EAC (right > left) and radiating to the bilateral face	None
	M	26	29	None	Deep inside the left EAC and radiating to the ipsilateral maxillary teeth	None
	F	15	23	Amitriptyline, carbamazepine, gabapentin, and local anesthetic blocks	Bilateral, deep inside; none deep inside the EAC (left > right)	
	M	65	67	Amitriptyline	Deep inside the right EAC	Middle ear infection, vertigo, deafness, and perforation of the tympanic membrane
	F	42	42	Pregabalin	Deep inside the left EAC and radiating to the jaw and neck	Middle ear infection
	F	53	55	Paracetamol	Deep inside the right EAC, pinna and skin, posterior and caudal to the pinna, and radiating to the ipsilateral cheek toward the right eye	Tympanic membrane perforation and middle ear infection
	F	44	48	Ibuprofen, carbamazepine, and amitriptyline	Deep inside the left EAC	Middle ear infection
Chen et al. (15) 1 case	F	45	55	Carbamazepine	Deep inside the right EAC, auricle, and cheek	Right HFS
Ozer et al. (16)	F	35	37	Carbamazepine	Tragus and radiating to the maxillary region	None
Pirillo et al. (17) 1 case	F	21	40	Unknown	Deep inside the left EAC	None
Saers et al. (18) 1 case	F	15	24	Carbamazepine, gabapentin, amitriptyline, and anesthetic blocks	Deep inside the left EAC	None
Sakas et al. (19) 1 case	F	46	52	Carbamazepine	Right auditory canal, pinna, and the adjacent retromastoid area	Ipsilateral tinnitus and right-sided hearing loss
Younes et al. (20) 1 case	F	58	63	Gabapentin and carbamazepine	Right otofacial area, radiating to the tongue, deep in the jaw, and ear, associated with paresthesias in the right lower jaw	Positional vertigo and double vision

*EAC, external auditory canal; HFS, hemifacial spasm; TN, trigeminal neuralgia; GPN, glossopharyngeal neuralgia*.

### Imaging and Intraoperative Findings

**Figure 2:**
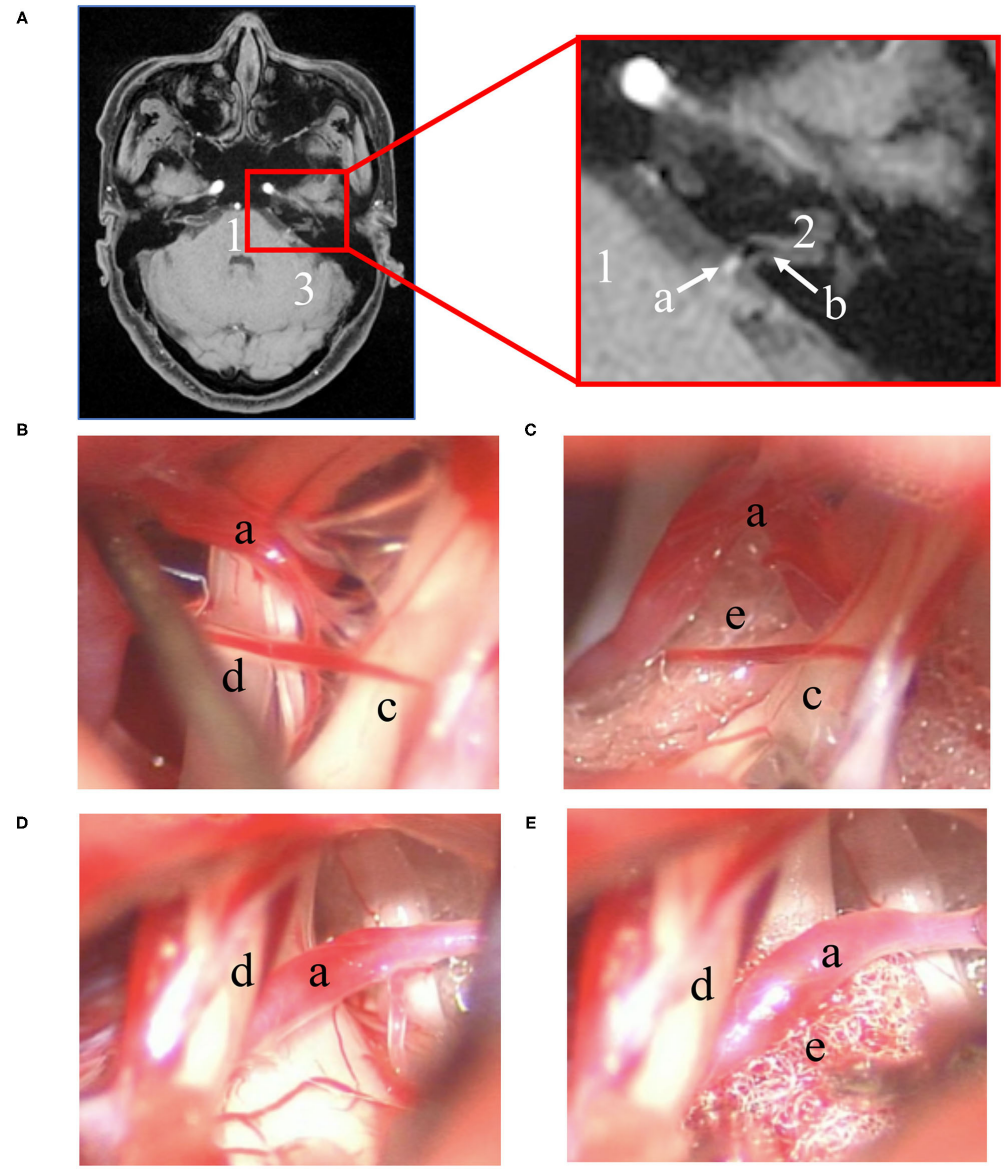
Preoperative imaging and intraoperative findings. **(A)** Left: transverse section position of the REZ of MRTA images. Right: magnification of the red box in the left image. Intraoperative microscope photograph of the MVD procedure: (1) one of the surgical procedures involved using a nerve stripper to stretch the facial nerve for direct visualization of the NI **(B)** and placing a small piece of Teflon between the NI and AICA **(C)**; (2) another procedure did not directly expose the NI **(D)**, and a small piece of Teflon was placed between the facial nerve and AICA **(E)**. (1: pons; 2: direction of the internal auditory canal; 3: left hemisphere of the cerebellum; a: AICA; b: the VII/VIII nerve complex; c: cranial nerve VII; d: cranial nerve VIII; e: Teflon).

Obvious neurovascular conflict (NVC) of the VII/VIII cranial nerve complex was clearly present in the preoperative MRTA image preoperatively. However, it is worth noting that not all the patients' offending vessels were preoperatively identified as the anterior inferior cerebellar artery (AICA), and some of their offending vessels were uncertain on MRTA imaging. In total, the main offending vessel was the AICA [17 of 23 patients (73.9%)], and the others were small vessels [6 of 23 patients (26.1%)]. As shown in Table 2, the relationship of the NVC with cranial nerves is as follows: (1) offending vessels run through the VII/VIII cranial nerve complex, (2) these offending vessels loop into the internal acoustic meatus, and (3) multiple vessels compress the cranial nerves at the root entry zone (REZ) (Figure 2, 1a).

**Table 2:** Pre- and intraoperative findings in NIN patients.

**References**	**No. of cases**	**Preoperative imaging findings**	**Offending vessel**	**Intraoperative relationships with offending vessels**	**Location of compression site**	**Direct visualization of NI**	**Surgical outcome**
Present cases	7	Vessels surrounding **(4)**, looping around **(1)**, and entering **(2)** the VII/VIII cranial nerve complex	AICA **(5)** and unidentified vessels **(2)**	Surrounding **(1)**, making contact with **(4)**, and entering **(2)** the VII/VIII cranial nerve complex	NVCs involving the VII/VIII cranial nerve complex were directly observed in all 8 cases	Visible (4) and none (3)	Excellent (2), facial paralysis (3), facial paralysis and dysacousia (1), and fair (1)
Inoue et al. (14)	1	None	AICA	Running between the VII/VIII cranial nerve complex	Under the flocculus to the vestibulocochlear nerve	None	Excellent
Onoda et al. (8)	1	Deformity of the VII/VIII complex	AICA	Surface contact with the VII/VIII cranial nerve complex	The vicinity of the internal auditory canal	None	Excellent
Chen et al. (15)	1	Small vessels around the facial nerve root	AICA	Looped into the VII/VIII cranial nerve complex	The lateral side of the facial nerve root	Definitively visible	Excellent
Ozer et al. (16)	1	None	AICA	Running between and adherent to the VII/VIII cranial nerve complex	The anterior segment of the facial acoustic bundle	Visible (intraoperative data)	Marked dysacousia and severe vertigo
Pirillo et al. (17)	1	A conflict between the VII/VIII cranial nerve complex and AICA	A branch of the AICA	Perforating recurrent branch running into the VII/VIII cranial nerve complex	Contact between the AICA lateral pontomedullary segment and the acoustic facial complex	Unknown	Excellent
Saers et al. (18)	1	The AICA approach to the VII/VIII cranial nerve complex	AICA	Bends upwards and runs vertically into the internal auditory canal and runs horizontally along the facial nerves	The left cerebellopontine angle at the level of the acousticofacial bundle	None	Excellent
Sakas et al. (19)	1	The AICA was extensively curved and was clearly compressing the VII/VIII cranial nerve complex	AICA	The concave part of the loop runs around and compresses the VII/VIII cranial nerves complex	The sensory branch of the VII cranial nerve	None	Excellent
Younes et al. (20)	1	None	AICA	Loop into the VII/VIII cranial nerve complex	A separate branch from the rest of the VIII cranial nerve	None	Excellent
Goulin Lippi Fernandes et al. (2)	8	Vessels surrounding **(3)**, loop entering **(2)**, looping around **(2)**, entering **(1)** the VII/VIII cranial nerve complex	AICA **(2)**, unidentified vessels **(4)**, and multiple vessels including the AICA **(2)**	Surrounding **(3)**, making contact with **(2)**, and entering **(3)** the VII/VIII cranial nerve complex	NVCs involving the VII/VIII cranial nerve complex were directly observed in all 8 cases.	None	Excellent **(4)**, hypoacusis **(3)**, and fair **(1)**

*AICA, anterior inferior cerebellar artery; NIN, nervus intermedius neuralgia; NI, nerve intermedius*.

Intraoperatively, there was an adhesive proliferated arachnoid membrane surrounding the vessels and VII/VIII nerve complex. Significantly, NVC involving the VII/VIII cranial nerve complex was directly observed during the operation. After careful sharp or blunt separation, six of 23 patients' (26.1%) NI was directly visualized under a microscope (Figure 2, 1b). This step was not achieved in the remaining patients (Figure 2, 1d). Subsequently, a small piece of Teflon was placed between the offending vessel and nerve (Figure 2, 1c,e), and MVD was performed successfully along the exposed VII/VIII nerve complex in all patients. Meanwhile, NVCs involving the trigeminal and glossopharyngeal nerves were entirely treated via MVD.

### Postoperative Outcome and Follow-Up

**Figure 3:**
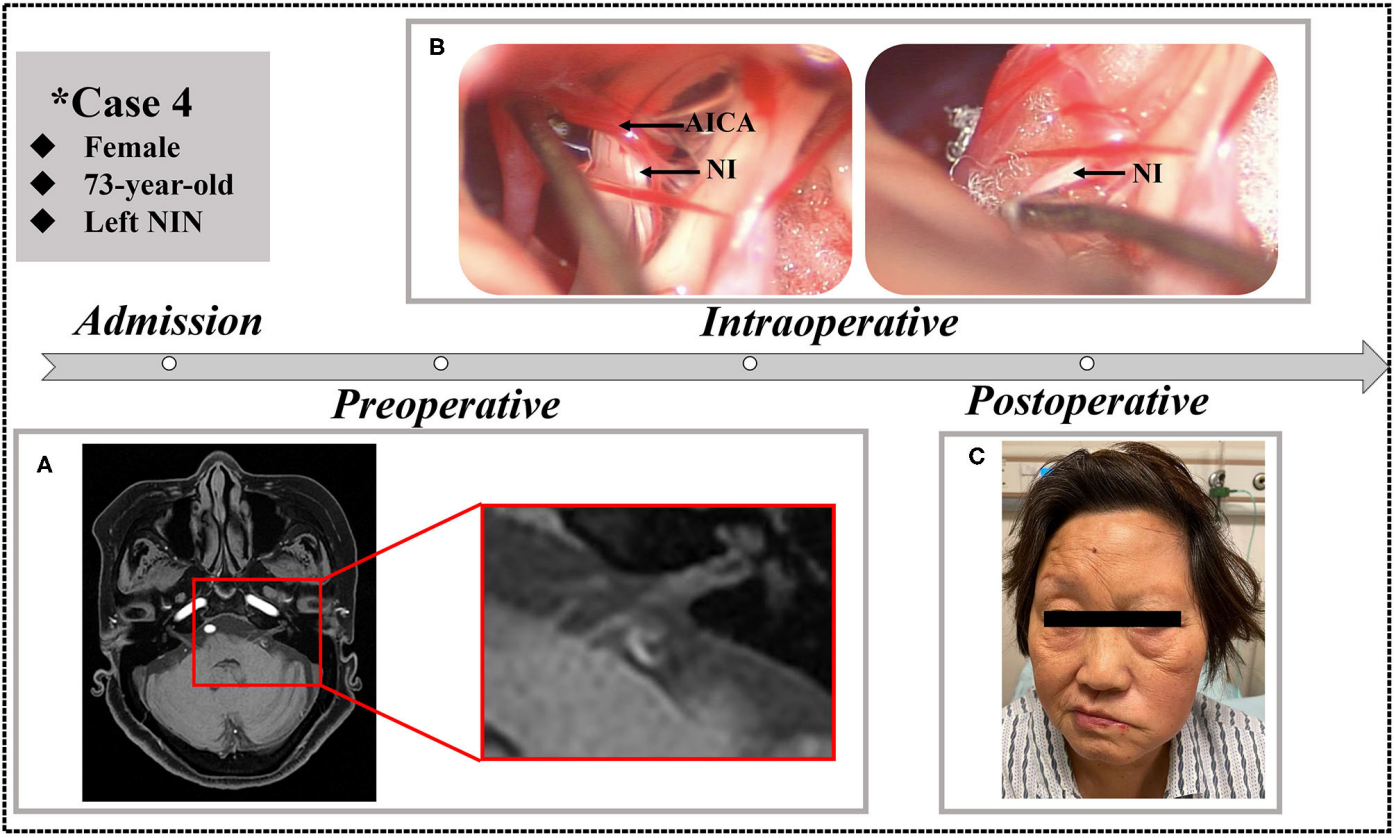
A 73-year-old female who underwent MVD in our clinic. **(A)** Preoperative MRTA shows the AICA entering the VII/VIII cranial nerve complex. **(B)** Separation to direct visualization of the NI during the MVD procedure. **(C)** The patient exhibited left facial paralysis postoperatively.

Postoperatively, the outcomes of our NIN patients are shown in Table 3. As shown in Figure 3, severe facial paralysis was observed in these patients from the first day after the operation to discharge (~2 or 3 weeks).

**Table 3:** Patients' outcomes.

**Patients**	**Follow-up months**	**Direct visualization of the NI**	**NIN symptom**			**Outcomes**		
			**Immediately postoperative**	**At discharge**	**6 months**	**Short duration**	**Duration till discharge**	**Permanent**
Case 1	12	Yes	Pain free	Pain free	Pain free	Facial paralysis and hearing loss	Facial paralysis and hearing loss	Facial paralysis and hearing loss
Case 2	9	None	Pain for 2 days	Pain improved	Pain free	Nausea and vertigo	None	None
Case 3	13	None	Pain free	Pain free	Pain free	Light vertigo	None	None
Case 4	16	Yes	Pain free	Pain free	Pain free	Facial paralysis and delayed wound union	Facial paralysis	Facial paralysis
Case 5	7	None	Pain for 1 week	Pain slight relapse	NIN relapse	Nausea	None	None
Case 6	8	Yes	Pain free	Pain slight relapse	Pain free	Facial paralysis numbs, nausea, and vertigo	Facial paralysis and numbs	Facial paralysis
Case 7	27	Yes	Pain improved	Pain improved	Pain free	Facial paralysis	Facial paralysis	Facial paralysis

In total, 21 of the 23 patients (91.3%) experienced an excellent pain-free status after the surgery and remained discharged. It is worth noting that one of our patients experienced immediate pain-free status after the operation and experienced a slight relapse after the first postoperative day but recovered before discharge. Another patient in the previous literature experienced relapse because of episodes of sigmoid sinus thrombosis. In addition, the complications of facial paralysis, dysacousia, or a combination of these conditions occurred in four of six patents (66.7%) who underwent direct visualization of the NI. No complications of hearing loss, vestibular dysfunction, taste abnormality, or dryness of the eye occurred in the remaining patients. Overall, the vast majority of patients recovered from NIN and reported complete pain-free status after 6 months of telephone or outpatient follow-up.

## Discussion

To the best of our knowledge, this study is the first to fully analyze whether it is necessary to perform blunt or sharp separation until achieving direct visualization of the NI during the MVD procedure. We found that NIN patients can benefit from MVD of the VII/VIII cranial nerve complex not only in our institutions but also in the literature records.

NIN is a rare cranial neuralgia disease, and fewer than 160 cases have been reported in the literature (2, 8). To date, clinicians have gradually reached a consensus that the main cause of NIN is NVC, but the specific pathophysiological mechanisms are still unknown (2, 3, 11). Given that trigeminal nerves, glossopharyngeal nerves, and vagus nerves constitute the sensory innervation of NI, a definitive diagnosis of NIN seems to be difficult (4). An accurate diagnosis of such neuralgia must first examine the internal auditory canal and the middle ear and other nerves driving the pain syndrome cited in otorhinolaryngology (8). In addition, the branch of the lesser/greater occipital nerve, the auriculotemporal branch of the trigeminal nerve, the tympanic branch of the glossopharyngeal nerve, and the auricular branch of the vagus nerve overlap in the area of the EAC (21). Therefore, when formatting a diagnosis of NIN, a differential diagnosis such as trigeminal neuralgia (TN) and glossopharyngeal neuralgia (GPN) must be taken into consideration. However, the absence of radiating pain in the trigeminal nerve distribution area and sore throat during swallowing, always accompanied by lacrimation and a taste disorder, increases the possibility of a NIN diagnosis (8, 22). In our case series, four of seven patients were preliminarily diagnosed with NIN mainly on the basis of pain caused by stimulation of the EAC wall. Based on the abovementioned clinical features, no definite diagnosis of NIN was made in the remaining three cases.

Although the NI is extremely thin and difficult to evaluate by neuroimaging, a diagnostic MRTA examination seems to be useful in that it helps neurosurgeons to preoperatively understand the relationship between nerves and vessels (2). A previous study reported that it was difficult to identify the branch of the facial nerve in up to 20% of cases (4), and the NI was identified in 75% of cases using 3.0-T MRI neuroimaging examination (8). At this point, modern examination techniques are expected to have significant utility in NIN diagnosis. Furthermore, preoperative imaging examination provides an objective basis for further surgical procedures. In our case series, MRTA was carried out to evaluate NVC in the NI region. The neurovascular relationship (VII/VIII complex nerves compressed by the AICA or unidentified vessels) of all seven cases was well-studied before the operation. The accuracy of MRTA imaging was confirmed to be consistent with the intraoperative findings.

To our knowledge, surgical treatment is beneficial in NIN that are refractory to medical treatment. Currently, two surgical options can be considered: sectioning or MVD of the NI (23). A previous retrospective study reported that sectioning of the NI is safe and yields no major complications (such as facial paralysis or deafness) (24). However, there are several limitations of such traditional procedure (25, 26): (1) the excellent outcome of a pain-free or pain-controlled status was not defined; (2) the average length of the patients' follow-up was not clear; (3) there was a high recurrence rate; and (4) the majority of patients experienced a variety of complications such as tinnitus, ear fullness, vertigo, increased lacrimation, or transient CN VII palsy. To address such problems, an alternative strategy was to section the sensory auricular branch of the facial nerve (27). Although such an entirely extracranial procedure minimizes the surgical risk (23), its effectiveness is not clear because the sensory auricular branch of the facial nerve is a disputed anatomic structure in nature. Thus, the recently proposed MVD strategy appears to be superior to the sectioning procedure. Considering the severity of refractory NIN patients' complaints, a recent case series study demonstrated that the benefits of MVD outweigh the associated complications regardless of surgical risk (2). To our knowledge, postoperative permanent hearing loss results from vestibulocochlear nerve injury caused by direct manipulation, ischemia of the cochlea caused by labyrinthine artery or AICA traction, or excessive drainage of cerebrospinal fluid (28). Therefore, we tried to avoid these improper operations and paid scrupulous attention to our MVD procedure.

Unfortunately, nearly half of our NIN patients experienced postoperative facial paralysis in our clinic. This triggered our thinking based on the notion that such complications can be avoided by a potential surgical improvement of the MVD procedure. We reviewed our MVD operation video and the medical records reported in the literature again. Interestingly, all patients with postoperative facial paralysis experienced direct visualization of the NI during the MVD procedure. We found thickening of the arachnoid membrane and adhesions around the VII/VIII nerve complex. To separate the NI from the VII/VIII nerve complex, the operator uses a nerve stripper, microsurgical scissors, or aspirator to repeatedly stretch the facial nerve over a period of time. Cranial neuralgia is essentially a deterioration of neighboring neural structures secondary to extrinsic compression (13). In the process of blunt or sharp separation, slight physical effects or careless stretching will cause serious injury to degenerative nerves. In addition, the average adherence of the NI to the facial nerve is 8 mm, the average length of the intermediate segment is 10 mm, and in most cases, the NI is a branch of the facial nerve (4). Therefore, it is even more difficult to separate the NI from the VII/VIII nerve complex. Furthermore, the results of 18 of 23 (78.3%) cases with satisfactory outcomes from MVD surgery were sufficient to show the treatment benefits. Consequently, there is no need to become overly concerned with directly dissecting out the NI since all obvious compressing vessels are separated from the VII/VIII cranial nerve complex.

The results of our study are subject to several significant limitations, including small cohort sizes, short follow-up time of 6 months, and the clinical benefits had been observed maybe due to a placebo effect. With respect to the literature review, the included reports were scattered over a broad range of years and therapeutic levels, had heterogeneous and inconsistent outcomes, and had an absence of formal statistical analysis. In spite of these shortcomings, we reviewed the presentation, management, and outcomes of surgically treated NIN at our hospital, which informs about the therapeutic benefits of MVD to NIN. At this point, this is a small but essential step forward in our understanding and developing an optimal surgical strategy of this rare neurosurgical disease.

## Conclusion

We have herein presented clinical evidence that surgical separation of the NI is technically difficult because it is firmly attached to the rest of the VII/VIII cranial nerve complex, and dissection to direct visualization presents a high risk of postoperative facial paralysis. In the course of the MVD procedure, there is no need to directly dissect out the NI while all compressing vessels are separated from the VII/VIII cranial nerve complex. Although MVD carries a surgical risk, the treatment benefits always outweigh the postoperative complications. Finally, our study certainly encourages further (preferably prospective) exploration of more objective and optimal surgical alternatives for MVD as well as NI dissection.

## Data Availability Statement

The raw data supporting the conclusions of this article will be made available by the authors, without undue reservation.

## Ethics Statement

The studies involving human participants were reviewed and approved by Ethics Committee in the Tongren Hospital, Shanghai Jiaotong University School of Medicine, Shanghai, China. The patients/participants provided their written informed consent to participate in this study. Written informed consent was obtained from the individual(s) for the publication of any potentially identifiable images or data included in this article.

## Author Contributions

RZ contributed to the conception or design of the work and drafted this manuscript. CZ and ZZ performed the statistical analysis and prepared the clinical data. XL supervised this study. All authors read and approved the final manuscript.

## Conflict of Interest

The authors declare that the research was conducted in the absence of any commercial or financial relationships that could be construed as a potential conflict of interest.
